# Current and emerging anti-angiogenic therapies in gastrointestinal and hepatobiliary cancers

**DOI:** 10.3389/fonc.2022.1021772

**Published:** 2022-10-10

**Authors:** Nadia Saoudi González, Florian Castet, Elena Élez, Teresa Macarulla, Josep Tabernero

**Affiliations:** Department of Medical Oncology, Vall d’Hebron University Hospital and Vall d’Hebron Institute of Oncology (VHIO), Barcelona, Spain

**Keywords:** anti-angiogenic, tyrosine kinase inhibitor, neoangiogenesis, gastrointestinal cancer, hepatobiliary tumour, hepatocellular carcinoma, colorectal cancer

## Abstract

Gastrointestinal tumours are a heterogeneous group of neoplasms that arise in the gastrointestinal tract and hepatobiliary system. Their incidence is rising globally and they currently represent the leading cause of cancer-related mortality worldwide. Anti-angiogenic agents have been incorporated into the treatment armamentarium of most of these malignancies and have improved survival outcomes, most notably in colorectal cancer and hepatocellular carcinoma. New treatment combinations with immunotherapies and other agents have led to unprecedented benefits and are revolutionising patient care. In this review, we detail the mechanisms of action of anti-angiogenic agents and the preclinical rationale underlying their combinations with immunotherapies. We review the clinical evidence supporting their use across all gastrointestinal tumours, with a particular emphasis on colorectal cancer and hepatocellular carcinoma. We discuss available biomarkers of response to these therapies and their utility in routine clinical practice. Finally, we summarise ongoing clinical trials in distinct settings and highlight the preclinical rationale supporting novel combinations.

## 1 Introduction

The process of angiogenesis was identified in 1971 as one of the key steps in cancer progression, and has been considered a hallmark of cancer since 2000 (1, 2). Angiogenesis is a pathway that implies the growth of new capillary blood vessels to maintain oxygen and nutrient supplies during tumour expansion. Cancer cells develop this angiogenic capacity *via* an “angiogenic switch” triggered by the synthesis and delivery of different positive signals that encourage angiogenesis, such as vascular endothelial growth factor (VEGF) that binds to the VEGF receptor (VEGFR) located on endothelial cells. More molecules participate in this delicate equilibrium, which is a balance between proangiogenic and anti-angiogenic signals crucial for the angiogenic switch (3). Soon after the discovery of the angiogenic pathway, efforts were made to develop treatments to block this process. This included large monoclonal antibodies such as bevacizumab and small tyrosine kinase inhibitors (TKIs) including sorafenib, sunitinib, and regorafenib, that have been approved in different tumour types (4).

**Figure 1:**
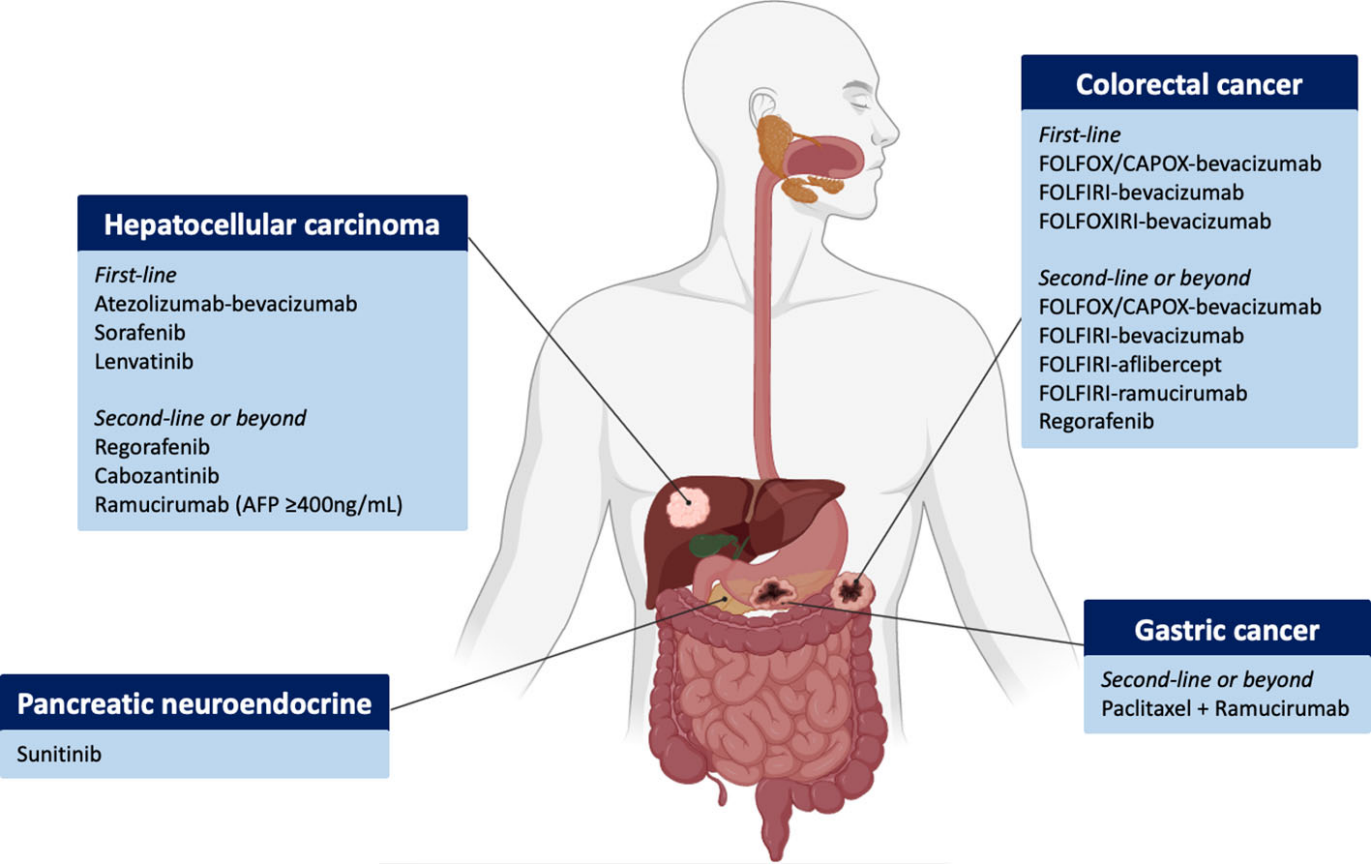
Overview of FDA-approved antiangiogenic agents across gastrointestinal and hepatobiliary tumours and disease settings. Figure generated with www.biorender.com.

Gastrointestinal cancers are drivers of cancer mortality worldwide. Colorectal cancer (CRC) is the second cause of cancer-related deaths, liver cancer the third, and stomach cancer the fourth (5). Therefore, there is a global concern and a need to generate more efficient diagnostic and therapeutic approaches to increase patient survival, with anti-angiogenics representing an attractive target in this setting. VEGF expression is associated with poor prognosis in colorectal, gastric and pancreatic cancer (6–8). The value of testing anti-angiogenic therapy in different gastrointestinal cancers has been established, and today different treatments and combination regimens are available for these tumours (**Figure 1**), as summarised in this review. Nevertheless, there is an unmet need for a better understanding of the mechanism of resistance as well as of optimal selection of those patients more likely to benefit from VEGF-targeted therapy, thus novel therapeutic strategies are also reviewed.

## 2 Angiogenesis in gastrointestinal cancer

Angiogenesis is essential for tumour progression and develops following an “angiogenic switch” (9–11). The onset of this event is dictated by the balance of pro- and anti-angiogenic factors (10, 11), which eventually leads to chronic activation of proangiogenic factors favouring the formation of new, morphologically aberrant blood vessels that will sustain tumour development and foster metastatic spread (9). Despite angiogenesis being universal to all cancers, tumours exhibit diverse patterns of neo-vascularisation which may influence response to therapy. An example of this heterogeneity is seen with pancreatic ductal adenocarcinomas (PDAC) that are generally hypovascularised and hypoxic owing to the high desmoplastic microenvironment, which may limit drug delivery (12). Conversely, hepatocellular carcinomas (HCC) are hypervascular tumours with a characteristic radiological pattern and a highly abnormal vessel architecture resulting from the overexpression of VEGF (13, 14). Similarly, neuroendocrine tumours have a rich vascular supply and a dense microvascular network which together constitute one of the most useful diagnostic characteristics of these tumours (15). In addition to the observed between-tumour type heterogeneity, substantial differences exist amongst tumours belonging to the same anatomic location. In CRC, the Consensus Molecular Subtype (CMS) 4 (mesenchymal subtype), which constitutes ~25% of all CRC, is characterised by high stromal infiltration, increased angiogenesis and is associated with a significantly worse prognosis (16). Likewise, gastric cancers classified as genomically stable are enriched in angiogenic pathways (17). These traits may help to explain, at least in part, the different patterns of response to anti-angiogenic therapies in distinct tumour types and amongst patients.

### 2.1 Signalling pathways in angiogenesis

Different mechanisms may lead to the formation of new blood vessels, such as the proliferation of pre-existing endothelial cells (“sprouting angiogenesis”), recruitment of endothelial progenitor cells to the tumoural microenvironment (“vasculogenesis”), remodelling of pre-existing blood vessels (“intussusceptive angiogenesis”) or formation of new vessels by tumoural cells independently of endothelial cells (“vascular mimicry”) (18). In addition, tumours may develop close to pre-existing mature blood vessels thus ensuring an adequate blood supply without the need for developing new vessels (referred to as “vessel co-option”) (19). While all of these mechanisms are known to contribute to tumour vascularisation in gastrointestinal tumours, sprouting angiogenesis remains the dominant mechanism and is triggered by multiple proangiogenic pathways.

The most potent angiogenic pathway in cancer is the VEGF signalling pathway (20), which is composed of five ligands (VEGF-A, -B, -C, -D and placental growth factor [PlGF]) and three receptors (VEGFR-1, -2 and -3). In gastrointestinal cancers, hypoxia mainly upregulates VEGF-A in tumour cells. This in turn binds to VEGFR-2 that is expressed on endothelial cells, leading to proliferation, vascular permeability and endothelial cell migration (10, 20). The mammalian fibroblast growth factor (FGF) signals through the FGF receptors 1-4 (FGFR 1-4) to mediate multiple functions including angiogenesis, cellular proliferation, invasiveness and enhanced metastasis. In addition to VEGF and FGF, many other pathways have been shown to be highly relevant in regulating angiogenesis in these tumour types, including the platelet-derived growth factor (PDGF) family, and the angiopoeitin family which bind to the tyrosine kinases TIE-1 and TIE-2 (20).

### 2.2 Mechanism of action of anti-angiogenic drugs

Anti-angiogenic therapies commonly used in gastrointestinal malignancies can be broadly categorised into monoclonal antibodies and TKIs. The former includes bevacizumab, which binds to VEGF-A, ramucirumab (which inhibits VEGFR-2) and aflibercept, a decoy receptor that binds to all isoforms of VEGF-A. TKIs are small-molecule compounds that inhibit a broad range of protein kinases. They include sorafenib, lenvatinib, regorafenib, cabozantinib and sunitinib, amongst others (**Figure 1**). The main targets of sorafenib are VEGFR-1, -2, -3, PDGFR, RAF and KIT; regorafenib and sunitinib have a similar inhibitory profile, as does lenvatinib that additionally targets FGFR-1, -2, -3 and -4. Cabozantinib is a potent VEGFR-2 and MET inhibitor.

**Figure 2:**
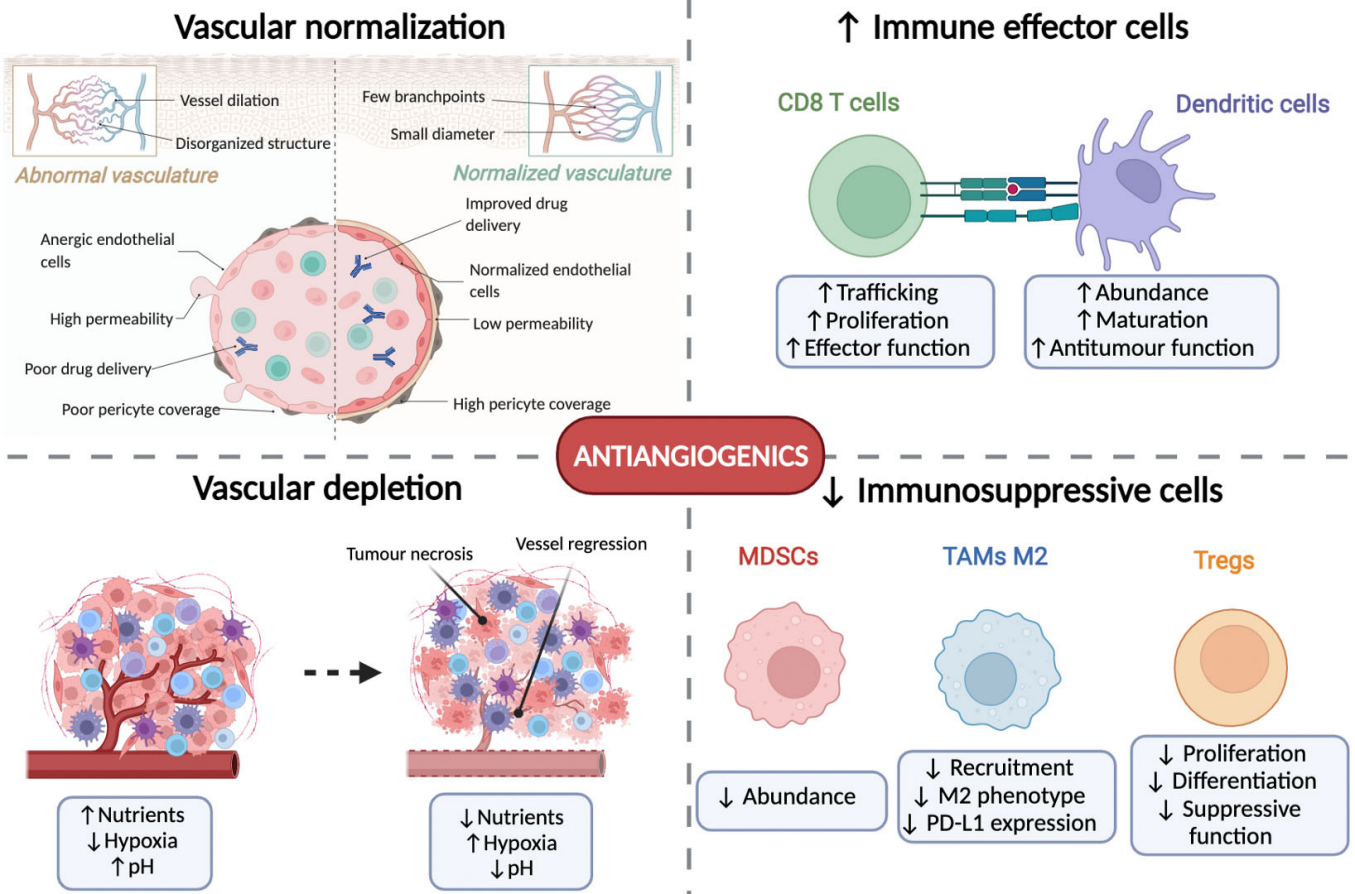
Mechanisms of action of antiangiogenic agents. Antiangiogenics exert their anti-tumoural effects *via* four axes. First, they induce normalisation of the tumour vasculature which improves drug delivery and oxygenation. Second, they induce vascular depletion which induces tumour starvation and necrosis. Thirdly, they favour an antitumour immune response by increasing the proliferation and activation of CD8+ T cells and dendritic cells. Finally, they decrease the presence and function of immunosuppressive cells including myeloid-derived suppressor cells (MDSCs), tumour-associated macrophages (TAMs) and T regulatory cells (Tregs). Figure generated with www.biorender.com.

Anti-angiogenic therapies may mediate antitumour effects in at least 4 different mechanisms (**Figure 2**). Firstly, the development of these therapies stems from the hypothesis that starving tumours by depleting them of blood vessels will induce necrosis and slow tumour progression (21). However, fostering a hypoxic and nutrient-deprived microenvironment may also result in treatment resistance and insufficient efficacy (22). This has led to the concept of vascular normalisation (23), understood as the resulting effect on the tumour vasculature of a judicious use of anti-angiogenic drugs that may balance the excess of proangiogenic factors and lead to a remodelling and pruning of tumour blood vessels to normalize the tumour vasculature (24). This, in turn, will improve drug delivery and foster a less hostile microenvironment, thus increasing the efficacy of combination partners (22).

Thirdly, increased attention has been placed on the immunomodulatory effects of anti-angiogenic therapies (**Figure 2**). Angiogenic modulating factors may alter the immune microenvironment through three established paths (25). First, VEGF can directly act on immune cells, leading to CD8^+^ T cell exhaustion, increased proliferation of T regulatory cells (Tregs), thereby fostering the expansion of myeloid-derived suppressor cells (MDSCs), and inhibiting the differentiation of monocytes to dendritic cells (DC) and decreasing DC maturation (26, 27). Secondly, the endothelium of tumour blood vessels creates a natural barrier for immune cells to infiltrate the microenvironment due to its lack of adhesion proteins (such as ICAM1 and VCAM1), as well as increased expression of proapoptotic molecules (FASL and galectin 1) and immunosuppressive molecules (PD-L1, PD-L2, TIM3 and IDO) (28). Finally, the hypoxic and acidotic tumour microenvironment also favours immunosuppressive changes, including the reprogramming of tumour-associated macrophages (TAMs) from an antitumour M1-like phenotype to a pro-tumoural M2-like phenotype, also decreasing the maturation and proliferation of DCs and increasing the proliferation of Tregs (26). All these factors will lead to a highly immunosuppressed microenvironment that could be potentially reversable with appropriate anti-angiogenic drugs.

These immunomodulatory effects have prompted the development of combinations of anti-angiogenic drugs with immune checkpoint inhibitors (ICIs). The rationale underlying these novel therapies is sound: ICIs increase the recruitment and/or activation of effector CD8^+^ T cells, DCs and natural killer cells and promote an antitumour M1 macrophage phenotype, while simultaneously decreasing the infiltration and activity of MDSCs, Treg cells and M2 macrophage polarisation (26, 29). Specific combinations in animal models further support this notion. Cabozantinib combined with anti-PD1 antibodies in syngeneic HCC mice models promoted the infiltration of neutrophils (30), and lenvatinib decreased the abundance of tumoural Tregs (31). Sorafenib specifically suppressed the activation of macrophages with an M2-like polarisation (32) and regorafenib favoured the infiltration of activated CXCR3^+^CD8^+^ T cells (33) and an M1-like macrophage polarisation (34). Similarly, sunitinib or antibodies blocking the VEGF/VEGF-R pathway in syngeneic colon mice models decreased the population of Tregs (35). Hence, combining therapies that target angiogenesis with immune stimulating agents represents a promising strategy that is being actively explored in clinical trials in many gastrointestinal, hepatobiliary and pancreatic tumours.

### 2.3 Mechanisms of resistance to anti-angiogenic drugs

Some tumours may be primarily resistant to anti-angiogenic drugs while others can develop mechanisms of resistance following drug exposure. This process of adaptation may undergo different sequential phases. In an early phase, tumours will upregulate alternative or redundant pro-angiogenic pathways that are not targeted by a specific drug, thus resulting in sustained angiogenesis despite optimal inhibition of the targeted pathway (36). Moreover, some tumours, such as PDAC, rely primarily on signalling pathways other than VEGF, leading to primary resistance to these inhibitors. In a later step, tumours adapt to hypoxia by promoting autophagy, which degrades cell components to promote survival in unfavourable conditions. In HCC, increased activation of mTOR or Akt pathways has been shown to trigger autophagy and cell survival when exposed to sorafenib, and can be overcome by combining sorafenib with autophagy inhibitors (37). The stress induced by antiangiogenic therapies stimulates inflammatory pathways and cytokines that lead to the recruitment of cells that favour angiogenesis, such as bone marrow-derived cells (BMDCs), myeloid-derived suppressor cells (MDSCs), endothelial progenitor cells, pericytes and cancer-associated fibroblasts (38, 39). In a late adaptation phase following exposure to antiangiogenic agents, tumours adopt different neovascularization modalities, including vessel co-option and vascular mimicry, which in turn may fuel metastatic spread and increase tumour aggressiveness (38).

An area of increasing interest and research is the significant heterogeneity of tumour endothelial cells which may contribute to resistance to anti-angiogenic drugs (40, 41). A single-cell analysis of endothelial cells following angiogenic inhibition has found that these cells can be broadly categorized into tip cells, transition and stalk-like cells. VEGF inhibition reduces all three subpopulations but has a particularly strong inhibitory effect on tip cells. In contrast, blockade of Dll4 promotes endothelial proliferation as well as tip cell markers (41). In liver tumours specifically, endothelial cancer cells show liver-specific gene expression signatures that are independent of the site of the original tumour, display distinct clusters that recapitulate tip-like and stalk-like characteristics and show stronger similarities to venous rather than endothelial cells (42). Furthermore, HCCs present endothelial cells that are reminiscent of fetal development, with a shared onco-fetal programme that is mediated in part by VEGF and NOTCH (43). In lung cancer, 13 different endothelial cell phenotypes have been described, including some subsets potentially involved in immune surveillance. This study further supports the notion that tip endothelial cells are particularly sensitive to antiangiogenic therapies (40).

## 3 Colorectal cancer

CRC is the third most frequently diagnosed cancer worldwide, and the second cause of cancer-related deaths (5). Unfortunately, approximately 20% of patients with CRC have metastatic spread at diagnosis (mCRC), and almost half of all patients will develop metastases during the course of the disease (44, 45). The incorporation of biological agents targeting two major pathways involved in mCRC such as the epidermal growth factor receptor (EGFR) targeted by panitumumab or cetuximab, and the VEGF signalling pathway suppressed by bevacizumab, aflibercept, ramucirumab and regorafenib have improved median overall survival (OS) to more than 30 months (46–48).

### 3.1 Clinical overview of anti-angiogenic drugs in mCRC

#### 3.1.1 Advanced disease: First-line setting

To define the optimal strategy treatment for patients with mCRC, is crucial to take into account the Eastern Cooperative Oncology Group (ECOG) performance status of the patient, the sidedness of colon tumour, molecular status of all *RAS* and *BRAF* genes, microsatellite status and resectability of metastatic disease, in addition to the patient’s preferences and toxicity of the treatments. According to European Society for Medical Oncology (ESMO) guidelines, combination of biological agents with FOLFIRI or FOLFOX chemotherapy is standard treatment for the first and second line setting in mCRC (45, 49–55).

Bevacizumab is a recombinant humanised monoclonal antibody targeting the VEGF ligand A (VEGF-A) and is approved for use in mCRC patients in the first and second lines of therapy. Over the last 20 years, multiple clinical trials have investigated the combination of bevacizumab with chemotherapy in this setting. The first phase III trial published in 2004 explored the combination of bevacizumab with irinotecan, bolus fluorouracil and leucovorin (IFL) *vs* IFL alone. Median OS was 20.3 months in the group that received IFL plus bevacizumab, compared with 15.6 months in the group given IFL plus placebo, corresponding to an HR for death of 0.66 (*P*<0.001) (56). Since then, the benefit of adding this monoclonal antibody to cytotoxic chemotherapy backbone regimens that contain either oxaliplatin or irinotecan, or both, or a fluoropyrimidine as monotherapy has been explored and demonstrated. **Table 1** summarizes the major clinical trials in this setting. In combination with first line oxaliplatin, the NO16966 trial demonstrated a benefit for the addition of bevacizumab to FOLFOX or CAPOX. There was a significant improvement in progression-free survival (PFS) with the addition of bevacizumab; however, the magnitude of benefit was smaller than expected, and neither median OS nor overall response rates (ORRs) were significantly higher in patients who received bevacizumab (53). The BECOME trial randomised patients with *RAS* mutant unresectable, liver-limited mCRC to receive bevacizumab plus mFOLFOX6 *vs* FOLFOX6 alone. This trial demonstrated higher ORR (55% *vs* 37%), median PFS (9.5 *vs* 5.6 months), median OS (25.7 *vs* 20.5 months), and complete (R0) resection rates (22.3% *vs* 5.8%) (57).

**Table 1:** Overview of selected phase III trials testing anti-angiogenic agents in mCRC.

Trial	Population	Treatment arms	N- patients	OS		PFS		ORR (%)	DCR (%)	Grade 3-4TRAEs (%)
				Median (mo)	HR (95% CI)	Median (mo)	HR (95% CI)			
Bevacizumab in first line										
Hurwitz et al. 2004	pre*RAS*	IFL + bevacizumab	402	20.3	0.66 (NA)*	10.6	0.54* (NA)	44.8*	NA	85
		IFL + placebo	411	15.6		6.2		34.8*		74
NO16966	pre*RAS*	XELOX/FOLFOX4 + bevacizumab	699	21.3	0.89 (0.76-1.03)	9.4	0.83 (0.72-0.95)*	47	NA	80
		XELOX/FOLFOX4 + placebo	701	19.9		8.0		49		75
BICC-C	pre*RAS*	FOLFIRI + bevacizumab	57	28	NA	11.2	NA	57.9	NA	NA
		FOLFIRI	144	23.1		7.6		47.2		
Bevacizumab in first line frail patients												
AVEX	≥70 years old	Capecitabine + bevacizumab	140	20.7	0.79 (0.57-1.09)	9.1	0.53 (0.41-0.69)*	19*	74*	40
		Capecitabine	140	16.8		5.1		10*	58*	22
Bevacizumab vs anti-EGFR treatment first line												
CALGB/SWOG 80405	*KRAS* wt (initially all *RAS*)	CT + cetuximab	578	30	0.88 (0.77-1.01)	10.5	0.95 (0.84-1.08)	59.6	NA	NA
		CT + bevacizumab	559	29		10.6		55.2	NA	NA
FIRE-3	*KRAS* exon 2 wt	FOLFIRI + cetuximab	297	28.7	0.77 (0.62-0.96)*	10	1.06 (0.88-1.26)	62	80	64
		FOLFIRI + bevacizumab	295	25		10.3		58	87	71
PARADIGM	*RAS/BRAF* wt left/right colon	mFOLFOX6 + panitumumab	312 (left)	37.9	0.82 (0.68-0.99)*	13.7	0.98 (0.82-1.17)	80.2	NA	NA
		mFOLFOX6 + bevacizumab	292 (left)	34.3		13.2		68.6	
TRIBE	Independent status *RAS*/*BRAF*	FOLFOXIRI + bevacizumab	256	29.8	0.8 (0.65-0.98)*	12.3 9.7	0.77 (0.64-0.94)*	65*	90	NA
		FOLFIRI + bevacizumab	252	25.8				53*	86
Bevacizumab second line												
ECOG E3200	pre*RAS* PD after 1^st^ L CT FU + irinotecan	FOLFOX4 + bevacizumab	286	12.9	0.75 (NA)*	7.3	0.61 (NA)*	22.7	NA	75
		FOLFOX4	291	10.8		4.7		8.6	61
		Bevacizumab	243	10.2	NA	2.7	NA	3.3	NA
ML18147	Independent status *KRAS* 1^st^L CT + Bevacizumab	CT + bevacizumab	409	11.2	0.81 (0.69-0.94)*	5.7	0.68 (0.59-0.78)*	6	69	64
		CT	411	9.8		4.1		4	54	57
Aflibercept second line												
VELOUR	Advanced – 2^nd^ line	Independent status *KRAS* PD 1^st^L oxaliplatin based CT	FOLFIRI + Aflibercept	13.5	0.82 (0.71-0.94)*	6.9	0.76 (0.66-0.87)	19.8*	86	83.5
		Placebo	FOLFIRI + placebo	12.06		4.7		11.1*	65	62.5
Ramucirumab second line												
RAISE	Independent status *KRAS* PD 1^st^L Oxaliplatin + FU + Bevacizumab	FOLFIRI + ramucirumab	536	13.3	0.84 (0.73-0.98)*	5.7	0.79 (0.70-0.90)*	13.4	NA	79
		FOLFIRI + placebo	536	11.7		4.5		12.5	62
Regorafenib in refractory setting												
CORRECT	Independent status *KRAS;* refractory setting	Regorafenib	505	6.4	0.77 (0.64-0.94)*	1.9	0.49 (0.42-0.58)*	1	41	54
		Placebo	255	5		1.7		0.4	15	14

5-FU, fluorouracil; CI, confidence interval; CT, chemotherapy; DCR, disease control rate; HR, hazard ratio; IFL, irinotecan, bolus fluorouracil, leucovorin; L, line; mo, months; NA, not available; ORR, overall response rate; OS, overall survival; PD, progressive disease; PFS, progression-free survival; TRAEs, treatment-related adverse events. *Indicates statistically significant differences.

For patients who are not suitable for doublet chemotherapy, the combination of fluoropyrimidines plus bevacizumab has demonstrated superiority over fluoropyrimidine monotherapy. The phase III AVEX trial focused on elderly patients with mCRC (70 years-old or older), enrolling patients who were not candidates for a combination of oxaliplatin or irinotecan-based chemotherapy regimens. Patients were randomised to bevacizumab plus capecitabine *vs* capecitabine alone, and this study demonstrated that the combination regimen was well tolerated and significantly improved outcomes, with median PFS of 9.1 *vs* 5.1, respectively (HR 0.53; 95% CI, 0.41–0.69; *P*<0.0001) (58). Median OS was not significantly different between the two groups as the study was not sufficiently powered to detect such differences (20.7 months in the bevacizumab plus capecitabine group *vs* 16.8 months in the capecitabine alone group [HR 0.79, 95% CI 0.57–1.09; *P*=0.18]).

Unlike frail patients, there are also a group of fit patients with metastatic disease who will benefit from a high response rate, thus more intense chemotherapy backbones have been investigated. Phase II/III trials have explored the combination of FOLFOXIRI with or without bevacizumab *vs* doublet combinations with or without bevacizumab (59–63). A recent individual-patient data meta-analysis of these clinical trials was published, demonstrating that FOLFOXIRI plus bevacizumab significantly and meaningfully improves OS of patients with mCRC compared with bevacizumab-based doublets and offers advantages in PFS, ORR and R0 resection rate, albeit at the price of increased toxicity. In contrast to initial observations from the subgroup analysis of the TRIBE trial, no increased benefit was observed among patients with *BRAF*V600-mutant tumours in this meta-analysis (61). Thus, the use of FOLFOXIRI–bevacizumab should no longer be regarded as the first choice for patients with a *BRAF*V600E mutation, in whom the use of FOLFOX–bevacizumab is currently the upfront treatment option of choice.

The question of which biologic is preferable in first line treatment for all *RAS* wild-type (wt) mCRC was addressed in several phase III trials. In the phase III trial FIRE-3, patients with previously untreated *KRAS* wt mCRC (initially this trial recruited allcomers, however due to the emerging evidence for the negative predictive value of *KRAS* exon 2 mutations, a protocol amendment was submitted in October 2007 to limit the population) received FOLFIRI plus cetuximab or FOLFIRI plus bevacizumab (48). There was no significant difference in the primary endpoint of ORR (65.3% with cetuximab *vs* 58% with bevacizumab, HR 1.18, *P*=0.18). The median PFS was similar in both the groups (10 months in the cetuximab group and 10.3 months in the bevacizumab group [HR 1.06, 95% CI 0.88–1.26; *P*=0.55]). Surprisingly, when evaluating *KRAS* exon 2 wt patients, OS was significantly better in patients treated with cetuximab (OS 33.1 *vs* 25.6 months favouring cetuximab over bevacizumab, *P*=0.011).

CALGB 80405 was a large randomised phase III trial in which patients with previously untreated mCRC received either FOLFIRI or FOLFOX at enrolment and were then randomised to either bevacizumab, cetuximab, or both (46). Initially, as for the FIRE-3, this trial included patients unselected for *RAS* status, with an amendment restricting eligibility to patients with *KRAS* wt tumours. The findings demonstrated similar results across all four groups, suggesting that either chemotherapy backbone in combination with either an anti-EGFR or anti-VEGF therapy is an acceptable therapy option in patients with *RAS* wt tumours.

mCRC is a clinically and molecularly heterogeneous disease, which is partially explained by the anatomic location of the tumour, given that left and right-sided tumours are derive from different embryonic structures (64). Furthermore, the left and right colon also have physiologically distinct functions with different contacts and exposure to nutrients, and thus different microbiomes can be found from the proximal to the distal colon (65). Regarding these differences, studies have retrospectively investigated the correlation between laterality and response to treatment, concluding that sidedness of colon cancer is a predictive biomarker of response to biological agents. From a meta-analysis published in 2017 covering all first line studies, a significant predictive benefit was demonstrated for chemotherapy plus EGFR antibody therapy in patients with left-sided tumours (HR 0.75 [95% CI 0.67-0.84] and 0.78 [95% CI 0.70-0.87] for OS and PFS, respectively) (66–68). However, there was a trend, albeit no significant benefit for patients treated with chemotherapy with or without bevacizumab with right-sided tumours (HRs 1.12 [95% CI 0.87-1.45] and 1.12 [95% CI 0.87-1.44] for OS and PFS, respectively). Recent data presented at ASCO 2022 from the PARADIGM trial, that randomised patients with *RAS* wt mCRC to receive panitumumab plus mFOLFOX or bevacizumab plus mFOLFOX, demonstrated a clear benefit of anti-EGFR therapy for patients with left-sided colon cancer (OS 37.9 *vs* 34.3 months; HR 0.82, *P*=0.031) (69). Thus, for patients with left-sided *RAS* wt disease, a cytotoxic doublet plus an anti-EGFR antibody should be the treatment of choice. For patients with right-sided *RAS* wt disease or *RAS* mutant, cytotoxic combination with bevacizumab is the preferred option.

The combination of both VEGF and anti-EGFR treatments is not recommended for first-line therapy of mCRC in light of the results of the PACCE and CAIRO2 trials (70, 71).

Maintenance treatment is a therapeutic strategy that envisages a period of high-intensity chemotherapy, after which agents that are mainly responsible for cumulative toxicity are stopped, leaving patients with a more simple and non-toxic combination of treatments until progression disease. This approach differs from treatment interruption, in which drug withdrawal is permitted with treatment-free intervals. Maintenance is active and should be part of the mCRC treatment strategy, as active maintenance with fluoropyrimidines and bevacizumab has demonstrated improvement of PFS (but not OS) (72, 73).

#### 3.1..2 Advanced disease: Second line setting

Several anti-angiogenic agents have demonstrated efficacy in mCRC in the second line setting.

Aflibercept is a fully humanised recombinant fusion protein composed of a modified immunoglobulin domain of VEGFR-1 joined to another immunoglobulin domain of human VEGFR-2 and fused to a fragment crystallizable portion of a human immunoglobulin, thus providing complete blockade of angiogenesis by targeting VEGF-A, VEFGF-B, and PIGF (74). In 2012, in the absence of evidence of improvement of OS in the second line in mCRC after progression on a first line oxaliplatin-containing regimen, the VELOUR trial was initiated (52). This randomised phase III double-blind study randomised 1226 patients into two groups, aflibercept or placebo every 2 weeks plus FOLFIRI. Data demonstrated advantages in OS, PFS and RR of aflibercept combined with FOLFIRI *vs* chemotherapy alone. Prior treatment with bevacizumab was permitted. The results showed an OS benefit favouring the aflibercept group, with an OS of 13.5 *vs* 12.1 months (HR 0.817; *P*=0.0032), PFS of 6.9 *vs* 4.67 months (HR 0.758; *P*<0.0001), and an ORR of 19.8% *vs* 11.1% (*P*=0.0001) with aflibercept plus FOLFIRI compared with placebo plus FOLFIRI, respectively. The effects of aflibercept exhibited a consistent trend of improved OS and PFS in pre-specified subgroup analyses based on previous treatment with bevacizumab. This higher efficacy of aflibercept was associated with an expected increase in adverse effects, with grade 3 and 4 adverse events (AEs) reported in 85.3% and 62.5% of patients, respectively.

The TML18147 trial was a randomised phase III trial that assessed the efficacy of bevacizumab beyond progression in patients with mCRC who had received first line treatment with bevacizumab (54). In this study, patients received bevacizumab with chemotherapy or chemotherapy alone, and demonstrated an improvement in OS for patients in the bevacizumab plus chemotherapy group (11.1 *vs* 9.8 months, respectively; *P*=0.0062).

Ramucirumab is a human monoclonal antibody that targets VEGFR-2. The phase III RAISE study evaluated the efficacy and safety of ramucirumab in combination with second line FOLFIRI compared with FOLFIRI plus placebo in mCRC patients who had progressed during or after first line therapy with bevacizumab and FOLFOX, independent of *KRAS* status (50). In this trial, a total of 1,072 patients were randomised to FOLFIRI with or without ramucirumab, showing a significant improvement in both OS and PFS (13.3 *vs* 11.7 months and 5.7 *vs* 4.5 months, respectively).

The results of these phase III trials support the benefit of continuing VEGF inhibition following prior exposure to bevacizumab. No direct comparison has been done, however the effects across all studies are of a similar magnitude, therefore the selection of bevacizumab, aflibercept or ramucirumab should be based on evaluating the toxicity profile, the interval free of bevacizumab, patient’s preference, reimbursement policy of each country and previous anti-EGFR in all patients with *RAS* wt mCRC.

#### 3.1.3 Advanced disease: Refractory setting

Regorafenib is an oral inhibitor that blocks the activity of multiple protein kinases active in oncogenesis and the tumour microenvironment, with anti-angiogenic activity due to its dual-targeted VEGFR2 tyrosine kinase inhibition (75). The efficacy of regorafenib in the mCRC refractory setting was demonstrated in the CORRECT trial (76). This phase III trial explored the efficacy in terms of OS of regorafenib *vs* best supportive care in patients with mCRC who progressed on standard therapy. Previous anti-angiogenic treatment was permitted. Patients were randomised in a 2:1 ratio to receive either best supportive care plus oral regorafenib or placebo once daily. Median OS was 6.4 months in the regorafenib group *vs* 5.0 months in the placebo group (HR 0.77; *P*=0.0052).

TAS-102 is an oral combination of a thymidine-based nucleic acid analogue, trifluridine and a thymidine phosphorylase inhibitor, tipiracil hydrochloride. TAS-102 has demonstrated efficacy in terms of OS compared to best supportive care in patients with refractory mCRC (5.3 months with placebo *vs* 7.1 months with TAS-102; HR 0.68, 95% CI 0.58 to 0.81; *P*<0.001) (77). The combination of bevacizumab and TAS-102 was explored in a phase II trial which randomised patients to receive standard-dose TAS-102 with or without bevacizumab (78). Combination therapy was associated with a modest, although statistically significant, improvement in median PFS (4.6 *vs* 2.6 months, HR 0.45, 95% CI 0.29-0.72) and OS (9.4 *vs* 6.7 months, HR 0.55, 95% CI 0.32-0.94) and a higher ORR (67% *vs* 51%).

### 3.2 Predictive biomarkers of anti-angiogenic drugs in colorectal cancer

Despite the importance of anti-angiogenic treatment for targeting this critical pathway of the disease, not all patients with mCRC benefit from this treatment, and in addition, a large proportion of them present severe AEs. There is an unmet clinical need driving the search for biomarkers of response to anti-angiogenic therapy in mCRC. Nevertheless, established biomarkers to predict response to anti-angiogenic treatments in mCRC are yet to be identified, as the different biomarkers tested to date have failed to show clear clinical utility (79, 80).

Retrospective data suggest that hypertension could predict treatment efficacy of bevacizumab in patients with mCRC (81). Some studies are researching the role of imaging in the assessment of vascularity of mCRC by radiomics of MRI and CT scan, attempting to translate medical images into biological information about tumour angiogenic status (82). A post-hoc analysis of the VELOUR trial showed that patients with previous bevacizumab treatment showed higher levels of VEGF-A and PIGF, suggesting that it could be a mechanism of resistance, and a negative prognostic marker in these patients, without differences in OS or PFS regarding plasma levels of VEGF-A and PIGF (83). Other retrospective data support this prognostic role of plasma levels of VEGF-A, without implications in prediction of response to anti-angiogenic treatment (84–86). Furthermore, the pattern of histopathological growth may influence response to anti-angiogenic agents (87). Vessel co-option is implicated as a major mechanism of resistance to these therapies and could represent a simple yet valuable biomarker of response (88).

### 3.3 Novel therapeutic strategies targeting angiogenesis in colorectal cancer

As previous reviewed, bevacizumab, aflibercept, regorafenib and ramucirumab have significantly improved both PFS and OS of mCRC in different clinical settings, from first line to the refractory scenario. Novel antiangiogenic agents and innovate combinations have been developed in recent years.

Fruquintinib is a novel receptor TKI inhibiting VEGFR 1, 2 and 3. Safety of this novel molecule was evaluated in a phase Ib trial, enrolling Asian patients with refractory mCRC, showing a manageable toxicity profile with the dosage of 5 mg once daily for 3 weeks with a 4-week cycle, giving a disease control rate (DCR) of 83.3% and 16-week PFS of 65% (89). The phase III FRESCO trial was a multicentre Asian trial in which 416 patients were randomised using a 2:1 ratio to receive fruquintinib with best supportive care or placebo plus best supportive care (90). Patients who received previous VEGFR inhibitors were excluded. Significant improvements were seen in the active fruquintinib treatment arm compared with placebo for OS (9.3 *vs* 6.6 months; HR: 0.65; *P*<0.001), PFS (3.7 *vs* 1.8 months; *P*<0.001), ORR (4.7% *vs* 0.0%; *P*=0.01) and DCR (62.2% *vs* 12.3%; *P*<0.001). This benefit was independent of previous treatment with anti-angiogenic agents or molecular status. The global FRESCO-2 trial (NCT04322539) is ongoing to confirm the results of the phase III FRESCO trial conducted in China.

Microsatellite stable (MSS) mCRC patients do not respond to monotherapy immunotherapy as demonstrated in many clinical trials (91–94). This population represents 95% of all patients with mCRC. Different combinations of immunotherapy with cofactors are being tested to achieve a change of a cold immune microambient to a hot microambient. One promising combination explored is the association of anti-angiogenic treatment with immunotherapy, as the blockade of VEGF leads to vasculature normalisation, thus permitting tumour infiltration with effector immune cells and the maturation of DCs (95–97).

Lenvatinib is a multiple kinase inhibitor. It inhibits the three main VEGFRs, VEGFR1, 2 and 3, as well as FGFR1, 2, 3 and 4, PDGFR, c-Kit and the RET proto-oncogene. Combination of pembrolizumab and lenvatinib has demonstrated the activation of CD8+ T cells, reduction of TAMS, leading to tumour reduction in murine models (98). A phase II non-randomised trial explored the combination of pembrolizumab with lenvatinib in MSS mCRC, demonstrating an ORR of 22% and a median PFS of 2.3 months (99). An ongoing randomised phase III trial (NCT04776148) is comparing lenvatinib plus pembrolizumab to standard of care in refractory MSS mCRC patients.

## 4 Hepatocellular carcinoma

HCC is the third leading cause of cancer-related death worldwide and its incidence is increasing globally (5). Most patients will be diagnosed at or progress to advanced stages, where systemic therapies remain the only effective option (100). Anti-angiogenic therapies constitute the treatment backbone of advanced HCC and their combination with immunotherapies has provided unprecedented benefits to this population (100–102). However, this has not yet been translated into the intermediate and early disease settings, and is an area of active research (103).

### 4.1 Clinical overview of anti-angiogenic drugs in HCC

#### 4.1.1 Advanced disease: First line setting

Sorafenib is the first TKI to demonstrate increased survival in HCC. It was approved in 2007 based on the results of the SHARP trial, an international, placebo-controlled phase III trial that randomised 602 patients with advanced HCC (BCLC-C or BCLC-B stage not amenable to transarterial chemo-embolisation [TACE]) with preserved liver function and performance status of 0-2, to sorafenib or placebo (**Table 2**). Sorafenib increased OS (median 10.7 *vs* 7.9 months, HR 0.69, 95% CI 0.55-0.87) and time to radiologic progression (median 5.5 *vs* 2.8 months, HR 0.58, 95% CI 0.45-0.74). The median duration of treatment was 5.3 months and the overall incidence of treatment-related AEs was 80% (104). These results were further supported by the Asia-Pacific trial, a randomised, confirmatory phase III trial with a similar design that was performed in China, South Korea and Taiwan, and randomised 271 patients to sorafenib or placebo (105). Sorafenib increased OS (median 6.5 *vs* 4.2 months, HR 0.68, 95% CI 0.5-0.93) although the median survival times were less than in the SHARP trial, owing to the inclusion of more advanced patients, with a higher proportion of BCLC-C patients (95% *vs* 82%), extrahepatic spread (69% *vs* 53%) and worse performance status (ECOG PS1 69% *vs* 38%).

**Table 2:** Overview of selected phase III trials evaluating anti-angiogenic agents in advanced HCC.

Trial	Disease setting	Treatment arms	N patients	OS		PFS		ORR (%)	DCR (%)		Grade 3-4TRAEs (%)
				Median (mo)	HR (95% CI)	Median (mo)	HR (95% CI)				
IMbrave150	Advanced – 1^st^ line	Atezolizumab + bevacizumab	336	19.2	0.66 (0.52-0.85)*	6.9	0.65 (0.53-0.81)*	30*	74*		43
		Sorafenib	165	13.4		4.3		11*	55*		46
SHARP	Advanced – 1^st^ line	Sorafenib	299	10.7	0.69 (0.55-0.87)*	NA	NA	2	43*		45*
		Placebo	303	7.9				1	32*		32*
Asia-Pacific	Advanced – 1^st^ line	Sorafenib	150	6.5	0.68 (0.5-0.93)*	NA	NA	3.3	35		NA
		Placebo	76	4.2				1.3	16		NA
REFLECT	Advanced – 1^st^ line	Lenvatinib	478	13.6	0.92 (0.79-1.06)*	7.4	0.66 (0.57-0.77)*	24.1*	75.5		57
		Sorafenib	476	12.3		3.7		9.2*	60.5		49
COSMIC-312	Advanced – 1^st^ line	Atezolizumab + cabozantinib	432	15.4	0.90 (0.69-1.18)	6.8	0.63 (0.44-0.91)*	11	78		53.8
		Sorafenib	217	15.5		4.2		3.7	65		31.9
		Cabozantinib	188		NA	5.8	0.71 (0.51-1.01)	6.4	84		55.2
LEAP-002	Advanced – 1^st^ line	Lenvatinib + pembrolizumab	395	21.2	0.84 (0.71-0.99)	8.2 8.1	0.83 (0.71-0.98)	26.1			61.5
		Lenvatinib	399	19				17.5			56.7
Qin et al.	Advanced – 1^st^ line	Rivoceranib + camrelizumab	272	22.1	0.62 (0.49-0.8)*	5.6 3.7	0.52 (0.41-0.65)*	78.3			80.9
		Sorafenib	271	15.2				5.9*	53.9		52.4
Qin et al.	Advanced – 1^st^ line	Donafenib	334	12.1	0.83 (0.70-0.99)*	3.7	0.91 (0.76-1.08)	4.6	30.8		38
		Sorafenib	334	10.3		3.6		2.7	28.7		50
SUN1170	Advanced – 1^st^ line	Sunitinib	530	7.9	1.3 (1.13-1.5)*	3.6	1.13 (0.99-1.3)	NA	NA		82.1
		Sorafenib	544	10.2		3					74.2
BRISK-FL	Advanced – 1^st^ line	Brivanib	577	9.5	1.07 (0.94-1.23)	NA	NA	12	66		67
		Sorafenib	578	9.9				8.8	65		65
LIGHT	Advanced – 1^st^ line	Linifanib	514	9.1	1.05 (0.9-1.22)	NA	NA	13	NA		85.3
		Sorafenib	521	9.8				7			75
SEARCH	Advanced – 1^st^ line	Sorafenib + erlotinib	362	9.5	0.93 (0.78-1.11)	NA	NA	6.6	43.9*		64.9
		Sorafenib	358	8.5				3.9	52.5*		63.7
RESORCE	Advanced – 2^nd^ line	Regorafenib	379	10.6	0.63 (0.5-0.79)*	3.1	0.46 (0.37-0.56)*	11	65		50
		Placebo	194	7.8		1.5		4	36		17
CELESTIAL	Advanced – 2^nd^ line	Cabozantinib	470	10.2	0.76 (0.63-0.92)*	5.2	0.44 (0.36-0.52)	3.8	64		68
		Placebo	237	8		1.9		0.4	33		37
REACH-2	Advanced – 2^nd^ line	Ramucirumab	197	8.5	0.71 (0.53-0.95)*	2.8	0.45 (0.34-0.60)*	59.9	NA		
		Placebo	95	7.3	1.6	1		38.9		
Qin et al.	Advanced – 2^nd^ line	Apatinib	267	8.7	0.79 (0.62-1)*	4.5	0.47 (0.37-0.60)*	11	61		77
		Placebo	133	6.8	1.9			2	29		19

DCR, disease control rate; HR, hazard ratio; mo, months; N, sample size; NA, not available; ORR, overall response rate; OS, overall survival; PFS, progression-free survival; TRAEs, treatment-related adverse events. *Indicates statistically significant differences.

Since the approval of sorafenib, it became the standard comparator arm in all phase III trials, most of which led to disappointing results (106–109). Lenvatinib is the first TKI to have demonstrated non-inferiority in terms of OS compared with sorafenib (110). The REFLECT trial was a phase III, international, sorafenib-controlled study that randomised 954 advanced HCC patients without main portal vein thrombosis, less than 50% of liver occupation and absence of invasion of the bile duct, to lenvatinib or sorafenib. Lenvatinib showed non-inferiority in terms of OS (median 13.6 *vs* 12.3 months, HR 0.92, 95% CI 0.79-1.06) but did not achieve superiority. However, lenvatinib did show superior PFS (median 7.4 *vs* 3.7 months, HR 0.66, 95% CI 0.57-0.77) and ORR (24.1% *vs* 9.2%, odds ratio 3.13, 95% CI 2.15-4.56) according to investigator assessment using mRECIST (110, 111). The open-label design of the study may have influenced the unexpected differences in treatment duration and time to progression between the sorafenib and lenvatinib arms (112), although subsequent real-world studies have confirmed the efficacy of lenvatinib (113).

The combination of atezolizumab-bevacizumab has become the new standard of care first line treatment in advanced HCC (114, 115). The IMbrave150 trial was a phase III international, sorafenib-controlled trial that enrolled 501 patients randomised in a 2:1 ratio to atezolizumab-bevacizumab or sorafenib. The trial met its primary endpoint of OS, showing an increase of 5.8 months at final analysis (median 19.2 *vs* 13.4 months, respectively, HR 0.66, 95% CI 0.52-0.85) (116). Additionally, the combination improved PFS (median 6.9 *vs* 4.3 months, HR 0.65, 95% CI 0.53-0.81) and ORR (30% *vs* 11%, *P*<0.001) (116). Treatment-related grade 3-4 AEs were observed in 43% of the patients in the atezolizumab-bevacizumab arm and 46% of the patients in the sorafenib arm. Importantly, five fatal upper gastrointestinal bleeding events were observed in the experimental arm, which were attributed to bevacizumab (116). Two previous phase II trials testing bevacizumab monotherapy had shown an increased risk of upper gastrointestinal bleeding in 7-11% of patients (117, 118). To mitigate this risk, a mandatory esophagogastroduodenoscopy had to be performed in the 6 months prior to enrolment and any varices had to be treated per local standard of care (114). A similarly designed phase III trial was reported in China and evaluated the combination of sintilimab, a programmed cell death protein 1 (PD-1) inhibitor, with IBI-305, a bevacizumab biosimilar. The trial randomised 571 patients to sintilimab-bevacizumab biosimilar or sorafenib in a 2:1 ratio and showed an improvement in OS (median not reached *vs* 10.4 months, HR 0.57, 95% CI 0.43-0.75) and PFS (median 4.6 *vs* 2.8 months, HR 0.56, 95% CI 0.46-0.7) (119). Both of these trials have demonstrated the efficacy of combining anti-VEGFA antibodies with ICIs.

The results of the COSMIC-312 trial, a phase III trial that tested the combination of cabozantinib and atezolizumab (120), enrolled 877 patients who were randomly assigned to the combination, cabozantinib or sorafenib in a 2:1:1 ratio. The dual primary endpoints are OS and PFS. The interim analysis demonstrated an improvement in PFS in the modified intention-to-treat population comprising the first 372 randomised patients (median 6.8 *vs* 4.2 months, HR 0.63, 95% CI 0.44-0.91), however, no improvement in OS was observed in the intention-to-treat population (median 15.4 *vs* 15.5 months, HR 0.9, 95% CI 0.69-1.18). The combination of TKIs with immunotherapies has been recently explored in two additional trials (121, 122). The LEAP-002 trial is an international, phase III, randomized, double blind study that enrolled 794 patients with advanced HCC and were randomly assigned to the combination of lenvatinib-pembrolizumab (N=395) or lenvatinib alone (N=399). The trial did not reach the prespecified threshold for none of the dual primary endpoints (OS and PFS). However, the median OS for the combination arm was the longest survival reported to date in the first-line setting (21.2 months). Importantly, this data further supported the role of lenvatinib monotherapy in this setting, with a median OS of 19 months (121). A second trial reported at ESMO 2022 was the combination of camrelizumab, an anti-PD1 monoclonal antibody, and rivoceranib, a VEGFR2-TKI, in the first-line setting. This was an international, phase III, open-label study that compared the combination to sorafenib (122). The dual primary endpoints were PFS and OS. The trial met it’s endpoints and showed a significant increase in OS (median 22.1 vs 15.2, HR 0.62 95% CI 0.49-0.8) and PFS (median 5.6 vs 3.7, HR 0.52 95% CI 0.41-0.65), as well as an increase in ORR (25.4 vs 5.9%). Despite these encouraging results, the combination will have to be tested in other populations as most of the included patients were Chinese. Furthermore, the open-label design of the study led to a high number of consent withdrawals in the control arm, which will have to be explored to understand its potential impact on the study results.

In China, the TKI donafenib has proved to be superior to sorafenib in the first line setting of advanced HCC (123).

#### 4.1.2 Advanced disease: Second line setting

Three TKIs have demonstrated improved outcomes in the second line setting after progression on sorafenib, regorafenib (124), cabozantinib (125) and ramucirumab in patients with alpha-feto protein (AFP) levels ≥400 ng/mL (126). The RESORCE trial was an international, phase III, double-blind, placebo-controlled trial that randomised 573 patients to regorafenib or placebo and was stratified based on region, performance status, macrovascular invasion, extrahepatic spread and AFP levels (124). Importantly, only patients who had previously tolerated sorafenib, defined as patients who had received ≥400 mg/day for ≥20 of the last 28 days of treatment, were included. Regorafenib significantly improved OS compared to placebo (median 10.6 *vs* 7.8 months, HR 0.63, 95% CI 0.5-0.79) and PFS assessed by mRECIST (median 3.1 *vs* 1.5 months, HR 0.56, 95% CI 0.37-0.56) (124). The rate of grade 3-4 treatment-related AEs was 50% in the regorafenib arm compared with 17% in the placebo arm.

The CELESTIAL trial had a similar design to the RESORCE trial although prior tolerance to sorafenib was not mandatory and patients could have progressed on up to two lines of systemic treatment. The trial randomised 707 patients in a 2:1 ratio to cabozantinib or placebo, stratified by region, macrovascular invasion, extrahepatic spread and disease etiology (125). It met its primary endpoint of OS (median 10.2 *vs* 8 months, HR 0.76, 95% CI 0.63-0.92) and showed a significant prolongation of PFS (5.2 *vs* 1.9 months, HR 0.44, 95% CI 0.36-0.52). Therefore, cabozantinib is the only TKI with evidence of efficacy in HCC following two prior lines of systemic treatment.

Ramucirumab was initially tested in the phase III REACH trial in the second line setting of advanced HCC following progression to sorafenib (127). Despite the trial being negative, an exploratory subgroup analysis showed significant benefit in patients with a baseline AFP level of ≥400 ng/mL. Hence, the REACH-2 study was designed as an international, phase III, double-blind, placebo-controlled trial and randomised 292 patients with an AFP level ≥400 ng/mL to ramucirumab or placebo (126). Ramucirumab increased OS (median 8.5 *vs* 7.3 months, HR 0.71, 95% CI 0.53-0.95) and PFS (2.8 *vs* 1.6 months, HR 0.45, 95% CI 0.34-0.6). A pooled analysis of all patients with baseline AFP levels ≥400 ng/mL (*N*=542) in both REACH and REACH-2 trials confirmed the survival benefit (median 8.1 *vs* 5 months, HR 0.69, 95% CI 0.57-0.84) (126).

In China, the VEGFR-2 inhibitor apatinib has improved survival in the second or third line settings of advanced HCC following treatment with sorafenib or FOLFOX (128).

### 4.2 Early and intermediate setting

The use of anti-angiogenic therapies in earlier settings of HCC have so far provided disappointing results. In the early setting, the only phase III trial to have tested TKIs as adjuvant therapy is the STORM study, an international, phase III, double-blind, placebo-controlled trial that enrolled 1114 patients with HCC suitable for local treatment (either ablation or resection) and a high or intermediate risk of recurrence (defined as tumours >2 cm or vascular invasion or satellites) to either adjuvant sorafenib for 4 years or placebo. No difference was observed in the primary endpoint of recurrence-free survival (median 33.3 *vs* 33.7 months, HR 0.94, 95% CI 0.78-1.13) or in OS (median not reached, HR 0.995, 95% CI 0.76-1.3) (129).

In the intermediate setting, three TKIs have been tested in combination with TACE in four phase III trials (130), namely, sorafenib (131, 132), brivanib (133) and orantinib (134). Unfortunately, none of these trials demonstrated an OS benefit compared with TACE alone. Whilst all four trials had a similar design, the primary endpoints were different: BRISK-TA (133) and ORIENTAL (134) trials assessed OS, while the trials testing sorafenib used time to progression (131) or PFS (132). Despite these discouraging results, the TACTICS trial was recently published, testing the combination of sorafenib initiated 2-3 weeks before TACE compared with TACE alone (135). This was a phase II, open-label, multicentre trial that enrolled 156 patients with a co-primary endpoint of OS and PFS. However, the definition of progression in this trial was unconventional and included untreatable tumour progression, transient deterioration to Child-Pugh C or appearance of vascular invasion/extrahepatic spread. The trial demonstrated a significant improvement in PFS (median 25.2 *vs* 13.5 months, HR 0.59, 95% CI 0.41-0.87) (135) but did not show any improvement in OS at the final analysis (median 36.2 *vs* 30.8 months, HR 0.86, 95% CI 0.61-1.22), casting doubts on the true value of this unconventional definition of progression (136).

Therefore, to date, no anti-angiogenic therapy is recommended in earlier settings of HCC.

### 4.3 Predictive biomarkers of anti-angiogenic drugs in HCC

The field of biomarker discovery in HCC is daunting and has so far led to disappointing results. The only available FDA-approved biomarker to guide treatment decision is AFP before initiating ramucirumab, based on the results of the REACH and REACH-2 trials (126, 127). No other biomarker has proven capable of predicting response to other anti-angiogenic therapies.

An exploratory analysis of 10 plasma markers (Ang2, EGF, bFGF, VEGF, sVEGFR-2, sVEGFR-3, HGF, s-c-KIT, IGF-2 and Ras) of patients enrolled in the SHARP trial found that despite that Ang2 and VEGF independently predicted survival in the entire cohort, none of the biomarkers assessed could predict response to sorafenib (137). In the sorafenib arm, high s-c-KIT and low HGF showed a trend towards enhanced survival (*P*-values of interaction 0.081 and 0.073, respectively) (137). Given the inherent difficulties of acquiring tissue specimens from advanced HCC patients, the same authors performed a thorough transcriptomic assessment of patients enrolled in the STORM trial, who were surgically resected and received sorafenib in the adjuvant setting (138). Tumour specimens from 188 patients were analysed by gene expression profiling, targeted exome sequencing, immunohistochemistry and fluorescence *in situ* hybridisation for VEGFA. None of the tested biomarkers, gene signatures or mutations predicted survival. A 146-gene signature was generated that could predict improved recurrence-free survival with sorafenib, although this has not been translated into the clinical setting due to lack of validation (138). Additionally, genomic variations of the *SCL15A2* gene, involved in drug transport, have been proposed as an additional biomarker of response to sorafenib (139). In line with this, a retrospective study found that the expression of OCT1 (another major player involved in sorafenib uptake) in the plasma membrane was associated with improved outcomes following sorafenib treatment (140). These studies highlight the importance of SLC transporters in sorafenib uptake and underline their possible impact on patient survival.

To identify potential biomarkers of response to regorafenib, an analysis of plasma from patients enrolled in the RESORCE trial was performed (141). The authors analysed 294 plasma proteins and 750 miRNAs. Additionally, next-generation sequencing of tumour tissue from 7 responders and 10 non-responders and expression of 770 genes involved in oncogenic and inflammatory pathways in 46 tumour tissues was performed (141). Decreased baseline plasma concentrations of five proteins (angiopoietin 1, cystatin B, the latency-associated peptide of transforming growth factor beta 1, oxidised low-density lipoprotein receptor 1 and C-C motif chemokine ligand 3) was associated with improved survival with regorafenib. Additionally, nine miRNAs were also associated with improved survival with regorafenib (MIR30A, MIR122, MIR125B, MIR200A, MIR374B, MIR15B, MIR107, MIR320, and MIR645) (141).

In a similar plasma analysis including VEGF, ANG2, FGF19, FGF21 and FGF23 of 407 patients included in the REFLECT study, a higher baseline level of FGF21 was predictive for longer OS with lenvatinib compared with sorafenib (*P*-value of interaction 0.0397) (142). Similarly, a plasma analysis of 674 patients included in the CELESTIAL trial did not identify any biomarkers predictive of response to cabozantinib (143). High levels of MET, HGF, GAS6, IL-8 and ANG2 and low levels of IGF-1 were associated with shorter survival in the placebo arm and this association was also observed for MET, IL-8, and ANG2 in the cabozantinib group (143).

More recently, an integrated molecular analysis was performed, comprising RNA sequencing, DNA sequencing and simple and multiplex immunohistochemistry of 358 patients included in the phase Ib GO30140 (144) and the phase III IMbrave150 trial (114, 116). This showed that pre-existing immunity, including the expression of a T effector transcriptomic signature and CD8+ T cell infiltration, predicted response to the combination of atezolizumab-bevacizumab, but not to sorafenib (145). Importantly, improved outcomes for the combination *vs* atezolizumab monotherapy was associated with high VEGFR-2 expression. Conversely, reduced benefit from the combination was associated with a low Treg/effector T cell ratio (145). These data highlight the synergistic effects of the combination of atezolizumab-bevacizumab and suggest several predictive biomarkers that will need validation in future trials.

### 4.4 Novel therapeutic strategies targeting angiogenesis in HCC

The breakthrough marked by the IMbrave150 trial with the atezolizumab-bevacizumab combination, has fuelled the development of multiple combinations that are being tested across all settings of this disease (102). Preclinical data strongly support combining anti-angiogenic drugs with immunotherapies and local treatments in the intermediate setting (101, 103). Local ablation or TACE increases the release of antigens, proinflammatory cytokines and proangiogenic factors (such as VEGF-A and HIF1) which promote an immune response that can be further sustained by increasing the activation of cytotoxic cells through immune checkpoint inhibition and decreasing the infiltration of immunosuppressive cells such as MDSCs and Tregs through the inhibition of angiogenesis (103). This constitutes the rationale for the design of trials combining TACE with durvalumab/bevacizumab (NCT03778957), atezolizumab/bevacizumab (NCT04712643) or pembrolizumab/lenvatinib (NCT04246177) (**Table 3**). More intriguingly, the outstanding survival outcomes observed with the atezolizumab and bevacizumab combination in the advanced setting, with a median OS of 19.2 months - which is similar to the expected survival of intermediate-stage HCC treated with TACE (100) - has sparked the development of two trials comparing standard TACE directly with systemic treatment (atezolizumab/bevacizumab [NCT04803994] or regorafenib/nivolumab [NCT04777851]) (**Table 3**).

**Table 3:** Overview of selected trials testing novel antiangiogenic agents and combinations in HCC.

Trial	Treatment	Phase	Setting	Enrolment target	Primary endpoint
NCT04102098 IMbrave050	Atezolizumab + bevacizumab	III	Early-adjuvant	668	RFS
NCT04639180	Camrelizumab + apatinib	III	Early-adjuvant	674	RFS
NCT04682210	Sintilimab + bevacizumab	III	Early-adjuvant	246	RFS
NCT03778957 EMERALD-1	TACE + durvalumab + bevacizumab	III	Intermediate	724	PFS
NCT04712643	TACE + atezolizumab + bevacizumab	III	Intermediate	342	OS TACE-PFS
NCT04246177 LEAP-012	TACE + pembrolizumab + lenvatinib	III	Intermediate	950	OS PFS
NCT05220020	TACE + lenvatinib	III	Intermediate	299	2y OS
NCT04803994 ABC-HCC	Atezolizumab + bevacizumab	III	Intermediate	434	TFTS
NCT04777851 RENOTACE	Regorafenib + nivolumab	III	Intermediate	496	PFS
NCT05320692	TACE + camrelizumab + apatinib	III	Intermediate	360	PFS
NCT05301842 EMERALD-3	TACE + durvalumab + tremelimumab +/- lenvatinib	III	Intermediate	525	PFS
NCT04194775	CS1003 + lenvatinib	III	Advanced – 1^st^ line	525	OS PFS
NCT04465734	HLX10 + HLX04	III	Advanced – 1^st^ line	477	OS PFS
NCT04344158	AK105 + anlotinib	III	Advanced – 1^st^ line	648	OS
NCT04560894	SCT-I10A + SCT510	III	Advanced – 1^st^ line	621	OS PFS
NCT04723004	Toripalimab + bevacizumab	III	Advanced – 1^st^ line	280	OS PFS
NCT04541173	Y90 TARE + atezolizumab + bevacizumab	II	Advanced – 1^st^ line	128	PFS
NCT05377034 STRATUM	SBRT + atezolizumab + bevacizumab	II	Advanced – 1^st^ line	176	ORR
NCT04976634	Lenvatinib + pembrolizumab + bezulfitan	II	Advanced – 1^st^ line	400	DLT Safety ORR
NCT04524871 Morpheus-Liver	Diverse drugs and combinations	I/II	Advanced – 1^st^ line	280	ORR
NCT04310709 RENOBATE	Regorafenib + nivolumab	I/II	Advanced – 1^st^ line	42	ORR
NCT04770896 IMbrave251	Atezolizumab + sorafenib/lenvatinib	III	Advanced – 2^nd^ line	554	OS
NCT04170556 GOING	Regorafenib + nivolumab	I/IIa	Advanced – 2^nd^ line	78	Safety
NCT04718909 REGSIN	Regorafenib + sintilimab	II	Advanced – 2^nd^ line	180	PFS
NCT04212221	MGD013 + brivanib	I/II	Advanced – 2^nd^ line	300	DLT Safety ORR
NCT03475953 REGOMUNE	Avelumab + regorafenib	I/II	Advanced – 2^nd^ line	482	ORR

DLT, dose-limiting toxicity; ORR, overall response rate; OS, overall survival; PFS, progression-free survival; RFS, recurrence-free survival; TACE, transarterial chemoembolization; TFTS, time to failure of treatment strategy; y, year.

Applying these combinations in earlier settings, when cure is still possible, is being eagerly pursued in phase III clinical trials. Three trials are currently exploring atezolizumab-bevacizumab (NCT04102098), camrelizumab/apatinib (NCT04639180) and sintilimab/bevacizumab (NCT04682210) in the postsurgical setting to decrease the risk of recurrence (**Table 3**). However, T-cell priming after the tumour was removed as this can be less efficient due to the close-to-non-existent tumour antigen burden (101). In addition, the high response rate achieved with new combinations could facilitate downstaging and improve tumour resectability when applied in the pre-surgical setting. Accordingly, a phase Ib study that enrolled 15 patients with unresectable HCC who received 8 weeks of neoadjuvant cabozantinib and nivolumab found that 13 patients ultimately underwent resection, 12 of whom had no residual tumour and 5 had major or complete pathological response. Importantly, none of the patients presented disease progression according to RECIST 1.1 (146).

Novel treatments and combinations are being explored in the advanced setting of HCC (**Table 3**). Lenvatinib-pembrolizumab constitutes one of the most promising combinations based on the phase Ib KEYNOTE-524 trial that enrolled 100 advanced HCC patients who had received no prior systemic treatment (147). The combination achieved an ORR of 46% according to mRECIST, with a disease control rate of 88%, a median OS of 22 months and median PFS of 9.3 months (147). This combination is currently being evaluated in a phase III trial (NCT03713593). To optimize the sequencing of TKIs and ICIs, the GOING trial (NCT04170556) is evaluating the priming effect of regorafenib monotherapy administered for 8 weeks prior to incorporating nivolumab into the regimen. Finally, the IMbrave251 trial (NCT04770896) is evaluating the combination of atezolizumab with sorafenib or lenvatinib in the second line following progression on atezolizumab-bevacizumab. These and other trials shown in **Table 3** are likely to change the treatment landscape of HCC in the near future.

## 5 Gastroesophageal cancer

Gastroesophageal cancers are a group of aggressive and highly lethal neoplasms. Gastric cancer represents the fifth most common cancer and the fourth most common cause of cancer-related death, while oesophageal cancer ranks seventh in terms of incidence and sixth in terms of mortality (5). Despite substantial advances over the last decade, prognosis remains poor, with an overall 5-year OS rate of 29% and 20% for gastric and oesophageal cancer, respectively (5). The pre-malignant form of oesophageal adenocarcinoma, known as Barrett’s oesophagus, expresses high levels of VEGFR2 (148). In gastric cancer, VEGF expression in tumour tissue or blood are correlated with prognosis, stage and risk of metastasis (149). Human epidermal growth factor receptor 2 (HER2) therapy and anti-angiogenic agents are the only two biological targeted therapies that have improved OS in patients with gastric or gastroesophageal junction adenocarcinoma.

There are currently no anti-angiogenic therapies approved for the treatment of oesophageal cancer. Small anti-angiogenic molecules such as sunitinib (in combination with paclitaxel or FOLFIRI) or sorafenib (in combination with docetaxel and cisplatin), erlotinib (with bevacizumab and neoadjuvant chemoradiation), apatinib (as maintenance treatment after chemo-radiation in localised oesophageal squamous cell carcinoma) or anlotinib (as monotherapy in the refractory setting) have shown limited or no efficacy in small phase II trials (150–154). Combinations of TKIs with immunotherapy are being evaluated (155). Bevacizumab was evaluated in combination with chemotherapy in two phase II trials, and was safe but with limited benefits (156, 157).

In gastric cancer, bevacizumab was evaluated in the AVAGAST phase III trial, comparing standard chemotherapy with or without bevacizumab, failing to show improvement in OS (158, 159). The phase III REGARD trial randomised patients with advanced gastric cancer to receive ramucirumab or placebo as second line treatment, with an OS of 5.2 *vs* 3.8 months, respectively (HR 0.776; *P*=0.047) (160). Following these results, the FDA approved ramucirumab for advanced gastric and gastroesophageal junction adenocarcinomas in 2014. The RAINBOW phase III trial compared weekly paclitaxel in combination with ramucirumab or placebo in patients refractory to a fluoropyrimidine plus platinum combination. Median OS was 9.6 months in the ramucirumab plus paclitaxel arm *vs* 7.4 months in the placebo plus paclitaxel group (HR 0.807; *P*=0.017), and paclitaxel plus ramucirumab became a recommended standard second line treatment for gastric cancer (161). RAINFALL was a global phase III trial that compared cisplatin plus capecitabine or 5-FU in combination with ramucirumab or placebo in the first line setting of patients with gastric or gastroesophageal junction adenocarcinoma (162). This trial demonstrated an improvement in PFS (5.7 *vs* 5.4 months; HR 0.75, *P*=0.011) but not in OS (11.2 *vs* 10.7 months; HR 0.96). Multiple trials are testing the combination of immunotherapy with ramucirumab, with promising signals of efficacy (163–165).

Apatinib is a small TKI that was tested in a Chinese phase III trial that compared apatinib with placebo in patients with refractory advanced gastric cancer, showing a statistically significant difference in OS (6.5 *vs* 4.7 months; *P*=0.0156) that led to its approval by the Chinese regulatory agency (166). The ANGEL trial (NCT03042611) is ongoing to confirm these results in the global population.

Other molecules such as aflibercept (in combination with FOLFOX), sorafenib (in combination with capecitabine plus cisplatin in the first line setting), sunitinib (in combination with FOLFIRI in second or third line) pazopanib (in combination with 5-FU plus leucovorin plus oxaliplatin) or fruquintinib (in combination with paclitaxel in second line treatment in China) have been tested in phase II trials, showing only marginal benefit in PFS (151, 167–171).

## 6 Neuroendocrine cancer

Neuroendocrine tumours (NETs) are a heterogeneous family of neoplasms that can arise from almost everywhere throughout the body, as they originate from the diffuse neuroendocrine system. NETs are rare (less than 7 new cases/100,000 inhabitants/year), however their incidence has increased over the last few decades (172). Well-differentiated NETs are characterised by rich vascularisation, with this phenomenon known as the “neuroendocrine paradox”, as the vascularisation is inversely related to the grade of aggressiveness of the tumour. NETs show high expression of PDGFR and c-Kit, demonstrating the importance of the angiogenesis pathway of these tumours (173). Sunitinib and surufatinib have shown activity in phase III trials *vs* placebo.

Sunitinib was tested in a phase III placebo-controlled trial of patients with advanced, well-differentiated pancreatic NETs (pNETs), in which patients could have received prior treatment (174). The study was discontinued early as a difference between placebo and sunitinib arm was observed benefiting patients in the active control arm. Median PFS was 11.4 months in the sunitinib group compared with 5.5 months in the placebo group (HR: 0.42; *P*<0.001). Posterior actualised data showed an improvement of median OS (38.6 *vs* 29.1 months in sunitinib *vs* placebo, respectively) (175). Surufatinib has shown benefit in a phase III placebo-controlled trial in advanced pancreatic NETs in a Chinese population (median PFS 10.9 months for surufatinib *vs* 3.7 months for placebo; HR 0.49; *P*=0.0011) (176). This agent has also reported benefit over placebo for extra-pancreatic advanced NETs in a Chinese population (176). Different phase II trials have tested multikinase inhibitors such as pazopanib, lenvatinib and cabozantinib, showing clinical activity in patients with NETs (177–181). Two ongoing phase III trials are evaluating axitinib (NCT01744249) and cabozantinib (NCT03375320).

## 7 Pancreatic and biliary tract cancer

Pancreatic cancer is a highly lethal disease with a rising incidence of 0.5-1% per year and is expected to become the second leading cause of cancer death by 2030 in the United States (182). Multiagent chemotherapy is recommended across all stages of the disease, either perioperatively in resectable/borderline resectable disease or to improve survival outcomes in advanced stages (183, 184). To date, only gemcitabine combined with albumin-bound paclitaxel (185) and FOLFIRINOX (fluorouracil, irinotecan, oxaliplatin, leucovorin) (186) regimens have demonstrated superiority over gemcitabine monotherapy in the first line metastatic setting, while FOLFOX (fluorouracil, oxaliplatin, leucovorin) (187, 188) and fluorouracil combined with liposomal irinotecan (189) have improved outcomes in the second line setting following a gemcitabine-based regimen. Most trials testing anti-angiogenic agents were performed before these combinations were approved and were combined with gemcitabine monotherapy in the first line setting. Bevacizumab was tested in two independent trials (**Table 4**). First, the CALGB80303 phase III trial randomised 602 advanced pancreatic cancer patients to gemcitabine monotherapy or combined with bevacizumab and showed no improvement in OS (median 5.8 *vs* 5.9 months, HR 1.05, 95% CI 0.88-1.24) (190). A second study tested the combination of bevacizumab with gemcitabine and erlotinib compared with gemcitabine and erlotinib alone and found a significant improvement in PFS (median 4.6 *vs* 3.6 months, HR 0.73, 95% CI 0.61-0.86) but no difference in OS (median 7.1 *vs* 6 months, HR 0.89, 95% CI 0.74-1.07) (191). Aflibercept was explored in a similarly designed phase III trial in combination with gemcitabine but was stopped early due to futility (192) and in a phase III trial with elpamotide, a peptide targeting VEGFR-2, the primary endpoint of OS was not reached when combined with gemcitabine (193). Additionally, several randomised phase II and III investigations have explored the use of different TKIs combined with gemcitabine including axitinib (194), sorafenib (195) and sunitinib (196), also failing to show any significant survival improvement over gemcitabine alone. More recently, the HCRN GI14-198 phase II trial tested ramucirumab in combination with a multiagent chemotherapy and randomised 86 patients diagnosed with treatment-naïve advanced pancreatic cancer to modified FOLFIRINOX combined with ramucirumab or placebo (197). The trial failed to improve outcomes in terms of PFS, ORR and OS (197). The reasons behind the failure of these trials are largely unknown, although novel therapies modulating the desmoplastic microenvironment and tumour stroma may help to enhance the clinical activity of anti-angiogenic therapies in this disease.

**Table 4:** Overview of selected phase II-III trials testing antiangiogenic agents in pancreatic and biliary tract cancer.

Trial	Disease setting	Treatment arms	N patients	OS		PFS		ORR (%)	DCR (%)	Grade 3-4 TRAEs (%)
				Median (mo)	HR (95% CI)	Median (mo)	HR (95% CI)			
CALGB 80803	Pancreatic – 1^st^ line	Gemcitabine + bevacizumab	302	5.8	1.04 (0.88-1.24)	3.8	0.86 (0.74-1.01)	13	54	NA
		Gemcitabine + placebo	300	5.9		2.9		10	44	NA
Van Cutsem et al.	Pancreatic – 1^st^ line	Gemcitabine + erlotinib + Bevacizumab	306	7.1	0.89 (0.74-1.07)	4.6	0.73 (0.61-0.86)*	14	62	74
		Gemcitabine + Erlotinib + Placebo	301	6		3.6		9	59	70
Kindler et al.	Pancreatic – 1^st^ line	Gemcitabine + axitinib	316	8.5	1.01 (0.79-1.31)	4.4	1.01 (0.78-1.30)	5	35	NA
		Gemcitabine + placebo	316	8.3		4.4		2	35	NA
BAYPAN	Pancreatic – 1^st^ line	Gemcitabine + sorafenib	52	9.2	1.27 (0.84-1.93)	5.7	1.04 (0.70-1.55)	19	65	88
		Gemcitabine + placebo	52	8		3.8		23	71	79
Rougier et al.	Pancreatic – 1^st^ line	Gemcitabine + aflibercept	271	6.7	1.17 (0.92-1.47)	3.7	1.02 (0.83-1.25)	NA	NA	77
		Gemcitabine + placebo	275	7.8		3.7		NA	NA	67
Reni et al.	Pancreatic – 1^st^ line	Gemcitabine + sunitinib	28	10.6	0.71 (0.4-1.26)	3.2	0.51 (0.29-0.89)	0	52	NA
		Gemcitabine + placebo	28	9.2		2		0	21	NA
Bergmann et al.	Pancreatic – 1^st^ line	Gemcitabine + sunitinib	52	7	1.06 (0.69-1.63)	2.7	1.06 (0.71-1.58)	7	75	NA
		Gemcitabine + placebo	54	8.5		3.1		6	67	NA
PEGASUS-PC	Pancreatic – 1^st^ line	Gemcitabine + elpamotide	100	8.4	0.87 (0.49-1.56)	3.7	NA	NA	60	NA
		Gemcitabine + placebo	53	8.5		3.8		60	NA	NA
HCRN GI14-198	Pancreatic – 1^st^ line	FOLFIRINOX + ramucirumab	42	10.3	NA	5.6	NA	18	NA	NA
		FOLFIRINOX + placebo	40	9.7		6.7		23	NA	NA
ABC-03	Biliary tract – 1^st^ line	Cisplatin + gemcitabine + cediranib	62	14.1	0.86 (0.58-1.27)	8	0.93 (0.65-1.35)	44*	78	NA
		Cisplatin + gemcitabine + placebo	62	11.9		7.4		19*	65	NA
Valle et al.	Biliary tract – 1^st^ line	Cisplatin + gemcitabine + ramucirumab	106	10.5	1.33 (0.96-1.86)	6.5	1.12 (0.9-1.4)	31*	81	85
		Cisplatin + gemcitabine + placebo	101	13		6.6		33*	78	76
		Cisplatin + gemcitabine + merestinib	102	14	0.95 (0.67-1.34)	7	0.92 (0.73-1.15)	20*	83	79
Van Gogh	Biliary tract – 1^st^ line	Vandetanib	56	7.5	NA	3.4	1.3 (0.86-1.96)	3*	25	NA
		Gemcitabine + vandetanib	57	9.3		3.7	1.3 (0.75-1.7)	19*	30	NA
		Gemcitabine + placebo	52	10.1	NA	4.9		14*	40	NA
Moehler et al.	Biliary tract – 1^st^ line	Gemcitabine + sorafenib	49	8.4	1.20 (0.75-1.93)	3	1.28 (0.81-2.02)	14	86	NA
		Gemcitabine + placebo	48	11.2	4.9	1.28 (0.81-2.02)		10	90	NA

CI, confidence interval; DCR, disease control rate; HR, hazard ratio; mo, months; N, sample size; NA, not available; ORR, overall response rate; OS, overall survival; PFS, progression-free survival; TRAEs, treatment-related adverse events. * Indicates statistically significant differences.

Biliary tract cancer refers to a spectrum of malignancies including cholangiocarcinoma and gallbladder adenocarcinoma (198). Their incidence is increasing globally, with a 5-year OS rate bordering 10%, and they represent ~2% of all cancer-related deaths worldwide annually (199). In advanced stages, the combination of cisplatin and gemcitabine has remained the established first line chemotherapy regimen for the past 12 years (200, 201), although this is likely to change given the improved survival observed with the addition of durvalumab in the TOPAZ-1 trial (202). The use of anti-angiogenic drugs has only been explored in phase II investigations and none of these combinations has reached later stages of development (**Table 4**). The largest of these trials was a randomised, phase II, three-arm trial exploring the combination of ramucirumab, merestinib or placebo with cisplatin-gemcitabine. The trial failed to meet its primary endpoint of PFS (median 6.5 *vs* 7 *vs* 6.6 months, ramucirumab *vs* placebo HR 1.12, 95% CI 0.9-1.4) (203). An exploratory analysis of mutations and gene expression signatures identified no predictive biomarkers (203). The ABC-03 trial was a randomised phase II trial that tested the combination of cediranib, an oral VEGFR-1, -2 and -3 inhibitor, with cisplatin and gemcitabine and showed no improvement in outcomes compared with placebo (204). In an exploratory biomarker analysis, circulating PDGFbb levels predicted benefit from cediranib (P-value of interaction 0.002) (204). Other trials that compared suboptimal chemotherapy regimens combined with sorafenib (205) or vandetanib (206) also failed to improve survival. New strategies, including the potential synergy of anti-angiogenic therapies when combined with immunotherapies and chemotherapy, are leading to novel combinations that could change the treatment landscape in the near future.

## 8 Discussion and future prospects

Anti-angiogenic therapies have been extensively evaluated across many gastrointestinal and hepatobiliary tumours. In some malignancies, such as HCC or CRC, these therapies provide unquestionable survival benefits either alone or combined with immunotherapy or chemotherapy, respectively. In others, such as oesophageal, biliary tract or pancreatic cancers, anti-angiogenics do not improve outcomes when combined with currently approved therapies. The development of novel anti-angiogenic strategies has stalled in recent years, partly due to the concurrent development of novel and highly effective drugs, especially immune-based therapies (207). However, whilst new anti-angiogenic drugs are not expected to enter the clinical setting in the near future, the use of approved anti-angiogenic therapies is likely to increase exponentially, owing to their highly synergistic effect with immunotherapies and other drug families (28). New combinations and applications in earlier disease settings is already being intensively explored in many diseases and will reshape the treatment scenario (101). Furthermore, recent studies have unveiled the heterogeneity of tumour endothelial cells and may support the use of patient-tailored antiangiogenic drug combinations to overcome this heterogeneity (40–42). Importantly, novel insights on resistance mechanisms support the combination of antiangiogenic drugs with distinct partners, such as inhibitors of the TGFß pathway, autophagy or CXCR4 (39). An intriguing area of research is the determination of the ideal dose of anti-angiogenic agents, especially when combined with other therapies. Until now, this has been based on the maximum tolerated dose in most cases, reflecting the belief that the higher dose of a drug will lead to a higher efficacy. Although this may be true for cytotoxic agents, this principle may not apply to anti-angiogenic drugs, where normalisation of blood vessels may be more important than vessel depletion to improve their synergistic effects (22).

Finally, the development of robust and validated biomarkers represents a clear unmet need in this field. It is unlikely that a unique biomarker common to distinct anti-angiogenic therapies will be identified owing to their diverse and heterogenous mechanisms of action, as well as inter and intra-tumoural heterogeneity. Furthermore, the utility of these potential biomarkers may be dependent on the combination partner, further emphasising the need to develop unique biomarkers for specific diseases and specific combinations. Despite these challenges, the importance of biomarker discovery in this field remains paramount. Radiomics, the study of vasculature in preclinical *in vivo* models and the analysis of new circulating angiogenic factors could shed some light on this unmet clinical need. Clinicians need additional tools to select the optimal therapy in each individual patient, given the increasingly complex treatment scenarios resulting from the continual approval of novel agents and combinations. Effective biomarkers would enable a better selection of patients and avoid unnecessary toxicities in those patients who are not expected to derive benefit from these agents. Therefore, adapting modern clinical trials to integrate biomarker-based objectives and pre-planned exploratory post-hoc analysis of baseline and on-treatment patient samples is fundamental and will become standard practice in the design of future trials.

## Author contributions

NS and FC have contributed equally to this work and share first authorship. All authors contributed to the article and approved the submitted version.

## Acknowledgments

The authors thank Sarah MacKenzie, PhD, for providing medical writing support.

## Conflict of interest

Author EE declares personal financial interests for consulting/advisory role and/or honoraria, travel grants and research grants from Amgen, Bayer, Hoffman-La Roche, Merck Serono, Sanofi, Pierre Fabre, MSD, Organon, Novartis and Servier. EE also declares institutional financial interests in the form of financial support for clinical trials or contracted research for Amgen Inc, Array Biopharma Inc, AstraZeneca Pharmaceuticals LP, BeiGene, Boehringer Ingelheim, Bristol Myers Squibb, Celgene, Debiopharm International SA, F. Hoffmann-La Roche Ltd, Genentech Inc, HalioDX SAS, Hutchison MediPharma International, Janssen-Cilag SA, MedImmune, Menarini, Merck Health KGAA, Merck Sharp & Dohme, Merus NV, Mirati, Novartis Farmacéutica SA, Pfizer, Pharma Mar, Sanofi Aventis Recherche & Développement, Servier, Taiho Pharma USA Inc. Author JT reports personal financial interest in form of scientific consultancy role for Array Biopharma, AstraZeneca, Bayer, Boehringer Ingelheim, Chugai, Daiichi Sankyo, F. Hoffmann-La Roche Ltd, Genentech Inc, HalioDX SAS, Hutchison MediPharma International, Ikena Oncology, Inspirna Inc, IQVIA, Lilly, Menarini, Merck Serono, Merus, MSD, Mirati, Neophore, Novartis, Ona Therapeutics, Orion Biotechnology, Peptomyc, Pfizer, Pierre Fabre, Samsung Bioepis, Sanofi, Scorpion Therapeutics, Scandion Oncology, Seattle Genetics, Servier, Sotio Biotech, Taiho, Tessa Therapeutics and TheraMyc. And also educational collaboration with Imedex, Medscape Education, MJH Life Sciences, PeerView Institute for Medical Education and Physicians Education Resource (PER).

The remaining authors declare that the research was conducted in the absence of any commercial or financial relationships that could be construed as a potential conflict of interest.

## Publisher’s note

All claims expressed in this article are solely those of the authors and do not necessarily represent those of their affiliated organizations, or those of the publisher, the editors and the reviewers. Any product that may be evaluated in this article, or claim that may be made by its manufacturer, is not guaranteed or endorsed by the publisher.
